# Immunohistochemical and biochemical characteristics of BSE and CWD in experimentally infected European red deer (*Cervus elaphus elaphus*)

**DOI:** 10.1186/1746-6148-5-26

**Published:** 2009-07-27

**Authors:** Stuart Martin, Martin Jeffrey, Lorenzo González, Sílvia Sisó, Hugh W Reid, Philip Steele, Mark P Dagleish, Michael J Stack, Melanie J Chaplin, Aru Balachandran

**Affiliations:** 1 Veterinary Laboratories Agency (VLA-Lasswade), Pentlands Science Park, Bush Loan, Penicuik, Midlothian, EH26 0PZ, UK; 2 Moredun Research Institute, Pentlands Science Park, Bush Loan, Penicuik, Midlothian, EH26 0PZ, UK; 3 VLA-Weybridge, Addlestone, Surrey, KT15 3NB, UK; 4 Animal Diseases Research Institute, Canadian Food Inspection Agency, Ottawa, Ontario, K2H 8P9, Canada

## Abstract

**Background:**

The cause of the bovine spongiform encephalopathy (BSE) epidemic in the United Kingdom (UK) was the inclusion of contaminated meat and bone meal in the protein rations fed to cattle. Those rations were not restricted to cattle but were also fed to other livestock including farmed and free living deer. Although there are no reported cases to date of natural BSE in European deer, BSE has been shown to be naturally or experimentally transmissible to a wide range of different ungulate species. Moreover, several species of North America's cervids are highly susceptible to chronic wasting disease (CWD), a transmissible spongiform encephalopathy (TSE) that has become endemic. Should BSE infection have been introduced into the UK deer population, the CWD precedent could suggest that there is a danger for spread and maintenance of the disease in both free living and captive UK deer populations. This study compares the immunohistochemical and biochemical characteristics of BSE and CWD in experimentally-infected European red deer (*Cervus elpahus elaphus*).

**Results:**

After intracerebral or alimentary challenge, BSE in red deer more closely resembled natural infection in cattle rather than experimental BSE in small ruminants, due to the lack of accumulation of abnormal PrP in lymphoid tissues. In this respect it was different from CWD, and although the neuropathological features of both diseases were similar, BSE could be clearly differentiated from CWD by immunohistochemical and Western blotting methods currently in routine use.

**Conclusion:**

Red deer are susceptible to both BSE and CWD infection, but the resulting disease phenotypes are distinct and clearly distinguishable.

## Background

Bovine spongiform encephalopathy (BSE) is one of a group of transmissible spongiform encephalopathies (TSEs), which include Creutzfeldt-Jakob disease in humans, chronic wasting disease (CWD) in deer and scrapie in sheep and goats. These invariably fatal neuro-degenerative disorders typically show lesions such as spongiform change, gliosis and deposition of the disease-associated form (PrP^d^) of the normal cellular prion protein (PrP^c^).

Contaminated meat and bone meal (MBM) within concentrated rations fed to cattle was the cause of the BSE epidemic in the United Kingdom (UK [1]) and elsewhere in Europe. Exotic ungulates were also exposed to this material resulting in cases of BSE in zoological collections [2,3], and it is likely that cervids in these collections were also exposed [4]. Similarly, contaminated rations could have also been fed to farmed and free ranging deer, the latter as a winter supplement. In March 2007, the European Union adopted a Commission Decision calling for a survey for TSEs in cervid populations, and monitoring of farmed and wild red deer began in the UK. Despite there not being any reported cases so far, the possibility that BSE infection occurred in deer prior to MBM bans cannot be ruled out as, on the other hand, several deer species are affected by CWD. One of those species is the elk or wapiti (*Cervus elaphus nelsoni*), which is closely related to the red deer (*Cervus elaphus elaphus*), the most frequently farmed cervid in the UK. Unlike other TSEs, CWD is the only TSE that is maintained in free-ranging animals with low population densities (2–3 deer per square mile), and surveillance data suggests an increasing prevalence of infection [5]. It is highly transmissible by natural routes of infection, so that the prevalence of infection can reach 100% in farmed herds [6], perhaps also influenced by the fact that, unlike sheep with scrapie, deer appear not to have any absolute genetic resistance to CWD [7].

Should BSE infection have been introduced to the UK red deer population, both the stability of the BSE agent through the species barriers and the high transmissibility of CWD, could account for spread and maintenance of BSE in free-living and captive UK deer. Due to the potential risk for human health from the consumption of contaminated deer products, it was deemed appropriate to determine the susceptibility or resistance of UK red deer to BSE and, if susceptible, to characterize the resulting disease phenotype in comparison with that of CWD in the same subspecies.

We previously described the susceptibility of UK red deer to BSE infection by intracerebral injection [8], and the present paper deals mainly with the comparative immunohistochemical (IHC) and Western blot (WB) features of experimental BSE and CWD infections in red deer. In addition, proof of concept is given that red deer are also susceptible to BSE by the oral route, albeit at high dose and apparently with a low attack rate and extended incubation period. In doing the experiments described here, we have also accrued a bank of positive control material to help future research and to be used as reference in the surveillance for BSE in red deer.

## Methods

### Experimental procedures

#### BSE experiments

Six one year-old red deer were intra-cerebrally (i.c.) inoculated with 0.5 ml of a 10% homogenate of a brain pool of five BSE-positive bovine brains (BBP12/92); this experiment has already been described [8]. A further six red deer aged 4 weeks were orally dosed via stomach tube with 25 g of another cattle BSE brain pool homogenate containing 10^3.5 ^RIII mouse (i.c./intraperiotoneal) units ID50/g; as with the BSE inoculum for i.c. challenge, this one also was provided by the TSE archive at VLA-Weybridge. Two and four negative control animals underwent identical procedures to the i.c and orally dosed deer, respectively, with sterile normal saline instead of brain homogenate. All experimental animal procedures were approved by the Moredun Research Institute Animal Experiments Ethical Committee and authorised under the UK Animals (Scientific Procedures) Act 1986.

#### CWD experiment

This was carried out by the Canadian Food Inspection Agency (Ottawa, Canada). Four European red deer were challenged with 5 g of a pooled brain homogenate from four elk with clinical CWD. The inoculum was administered in three doses three days apart and although it was not titrated, it gave an optical density reading of >3.5 (upper limit of measurement) in the Bio-Rad TsSeE test.

### Disease monitoring, post-mortems and tissue sampling

Deer challenged with BSE were clinically monitored daily and weighed monthly, until they reached a clinical end point established at the beginning of the experiment, as previously described [8]. After discussion and agreement, a similar approach was applied to CWD inoculated red deer. Biopsies of rectal mucosa were sequentially taken during the incubation period of both BSE and CWD inoculated animals; rectal biopsies may be used for pre-clinical diagnosis of TSEs in sheep [9] and in deer [10]. Deer were killed by barbiturate overdose and exsanguination. At post-mortem, an extensive range of tissue samples was taken and fixed in 10% neutral buffered formalin or frozen and stored at -80°C.

### Histopathology and IHC

Fixed samples were post-fixed in fresh 10% neutral buffered formalin and routinely processed for embedding in paraffin wax and light microscope examinations, either after staining with haematoxylin and eosin or immunolabelling for PrP^d^. The tissues examined by IHC from BSE inoculated deer have been described in detail previously [8], and included samples from the central and peripheral nervous systems, lymphoreticular system (LRS), and several other tissues. From CWD inoculated deer, eight brain areas and the medial retropharyngeal lymph node were available for IHC examinations.

The IHC method has been detailed elsewhere [11,12]. Briefly, tissue sections were subjected to antigen retrieval, peroxidase quenching and blocking of non-specific antigens prior to incubation with the primary antibody. This was carried out overnight at 27°C, and the subsequent steps of the IHC protocol were performed by a commercial immunoperoxidase technique (Vector-elite ABC kit; Vector Laboratories, Peterborough, UK) and finally counterstained with Mayer's haematoxylin. PrP primary antibodies used were BAR224 (CEA, Saclay, France), which recognizes amino acid residues (aa) 141–147 of ovine PrP [13], and 12B2 (CIDC, Lelystad, The Netherlands), recognizing aa 93–97 of ovine PrP [14]. Both antibodies have wide inter-species reactivity and were selected as the most specific and sensitive from a panel of antibodies tested against CWD positive and negative deer control tissues at the outset of the study.

### Western blot

Samples of medulla oblongata from BSE and CWD inoculated deer developing clinical disease and from non-inoculated controls were processed using the BioRad TeSeE universal WB, following the kit instructions. Briefly, 350 mg of tissue were ribolysed, purified, treated with proteinase K, and PrP^res^-concentrated. After heating, 20 mg equivalent of samples were loaded in duplicate lanes onto pre-cast 12% bis-tris gels (Bio-Rad laboratories Ltd. Bio-Rad House, Maxted Road, Hemel Hempstead, Hertfordshire, HP2 7DX.), and subjected to electrophoresis. The proteins were then transferred onto PVDF membranes, blocked (Bio-Rad blocking solution) and probed for 30 mins at room temperature with each of a panel of five antibodies: 6H4 (aa 156–164 [15]), F99 (aa 229–232 [16]), Sha31 (aa 156–163 [17]), P4 (aa 93–99 [18]), and 12B2. After washing, the membranes were incubated with the secondary antibody at room temperature, and with ECL substrate (Amersham Biosciences UK limited) for 45 s – 1 min, and the signal was detected with the Fluor-S MultiImager (Bio-Rad, Maxted Road Hemel Hempstead Hertfordshire HP2 7DX, UK).

The blots were assessed for molecular weights and proportions of the three PrP^res ^bands, and for the affinities for each PrP antibody. For comparison, brain samples from CWD positive and negative elk, BSE positive cattle and scrapie positive sheep were used.

### Discriminatory and scoring methods

The IHC "epitope mapping" and "PrP^d ^profiling", and the discriminatory WB allow differentiation between sheep BSE and natural and experimental scrapie in sheep and in other species. The IHC "epitope mapping" and the discriminatory WB are based on the availability of specific epitopes on PrP^d ^subjected to enzymatic attack "*in vivo*" (in intracellular lysosomes) and "*in vitro*" (by protease digestion), respectively [19,20]. It has been hypothesized that in experimental ovine BSE, epitopes between amino acids 93 and 97 of the PrP sequence are absent after enzymatic digestion, as truncation of the protein occurs further towards the C terminus, and therefore, these truncated forms of PrP^d ^are not labelled by 12B2 or P4. In contrast, in CWD and scrapie, the PrP^d ^molecule is truncated further towards the N terminus and the labelling with those antibodies is maintained after "*in vivo*" or "*in vitro*" digestion. "PrP^d ^profiling" is the most sophisticated technique for IHC strain differentiation: using a single antibody directed at the globular or C-terminal domains of the protein, this approach entails the subjective scoring (0–3) of different PrP^d ^types (intra-cellular, cell membrane bound and extracellular) across different areas of the brain to produce a graphic profile, which is representative of the IHC phenotype [11,12].

## Results

### Clinical disease

All six deer challenged i.c. with BSE developed clinical disease between 794 and 1260 days post-inoculation with a mean incubation period of 1027 days. A detailed description of the clinical signs was provided in an earlier report [8]. Briefly, affected deer showed variable degrees of ataxia, anorexia, circling and apparent blindness, together with failure of seasonal change of coat, weight loss and 'panic attacks'. In addition, one of six red deer orally dosed with BSE developed clinical disease 1740 days after challenge, and this animal presented with a short clinical duration of two days; the other five deer from this group remain healthy at the time of writing (65 months after challenge). Sequential rectal biopsies taken at five different time points from orally and i.c. inoculated deer were negative for PrP^d^.

All four deer orally challenged with CWD started to show behavioural changes between 577 and 586 days post challenge; these progressed to definite neurological disease between 742 and 760 days post-challenge (Table 1). Clinical signs were similar to the BSE challenged deer and included nervousness, weight loss, excessive salivation, roughness of coat, and progressive ataxia. All these CWD-inoculated deer showed PrP^d ^accumulation in the secondary follicles of rectal biopsies taken at 7 months post-infection.

**Table 1:** Experimental design and clinical data.

Inocula	Route		Deer					
			1	2	3	4	5	6
BSE	I.C	I.P	794	930	996	996	1060	1260
		C.D	21	100	105	77	85	22
	Oral	I.P	1740					
		C.D	2					
CWD	Oral	I.P	577	579	579	586		
		C.D	165	167	167	174		

Deer numbers, routes of infection, incubation period (I.P) and clinical duration (C.D) in days.

### Histopathology and IHC

#### Spongy change

**Figure 1:**
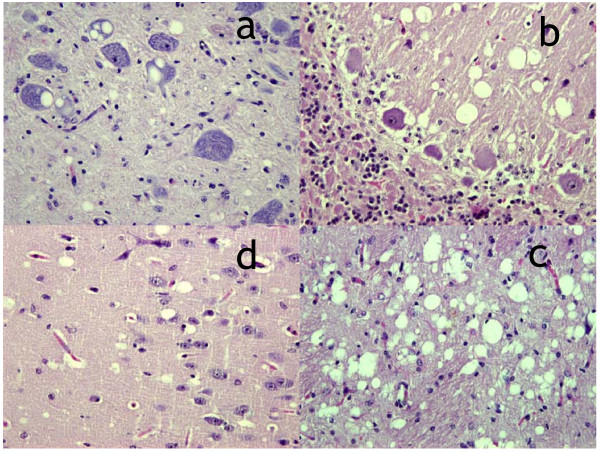
**BSE deer**. Vacuolation seen in the brainstem (a), the molecular layer of the cerebellum (b), the thalamus (c), and layers V and VI of the cerebral cortex (d).

In haematoxylin and eosin stained brain sections, spongiform change was widespread in all seven deer that developed clinical signs after BSE challenge regardless of the route. Vacuolation was seen in the neuronal perikaryon and neuropil of the brainstem, the molecular layer of the cerebellum, the thalamus, and the layers V and VI of the cerebral cortex (Fig 1). Spongy change was also observed in the same brain areas of the CWD-challenged deer; in these, intracytoplasmic vacuoles within neurones, although present, were less prominent than in the BSE challenged group. Otherwise no differences in pathological features were identified between the two groups of deer. Overall, spongiform changes and their distribution were similar to those reported in cattle with BSE [21], in sheep orally infected with BSE [22], and in CWD infected mule deer and elk [23].

**Figure 2:**
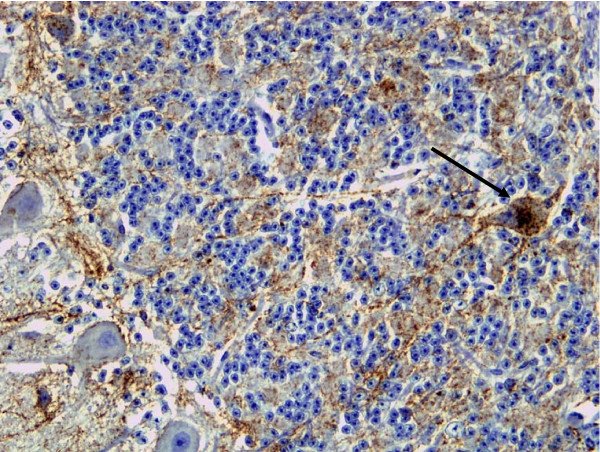
**BSE deer**. Grey matter PrP^d ^labelling of neuropil and intense perikaryonal labelling of a Golgi neuron in the granular cell layer of the cerebellum (arrow).

**Figure 3:**
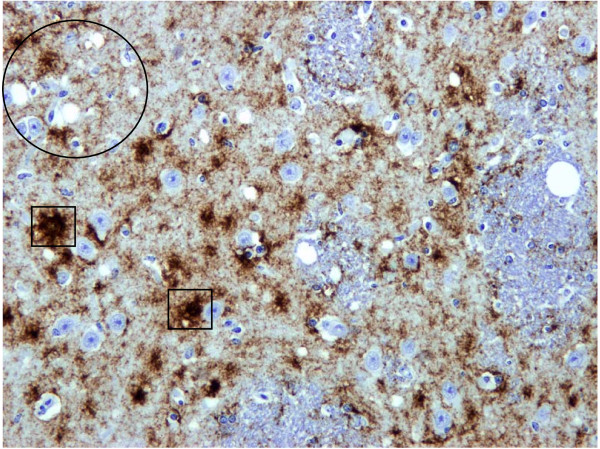
**CWD Deer**. Vacuolation (oval) and diffuse PrP^d ^accumulation is present in the grey matter of the striatum of the brain. Focal intense plaque-like accumulations of PrP^d ^(boxes) are also present.

**Accumulation of PrPd in the brain**. Widespread diffuse particulate labelling of the neuropil and intense intra-neuronal accumulations of PrP^d ^were observed throughout the brains of BSE affected deer in sections labelled with BAR224 antibody. Peri-neuronal labelling was prominent in the dorsal motor nucleus of the vagus nerve (DMNV) and in the *corpus striatum*. Intraneuronal granular accumulations were prominent in nuclei of the *medulla oblongata*, such as the accessory cuneate, spinal trigeminal and posterior olivary, but were also present elsewhere in the brain with the exception of Purkinje cells. Notably, there was also intense labelling of the Golgi neurones in the granular cell layer of the cerebellum (Fig 2), which has not been described in sheep or goat scrapie and which was absent in the CWD infected deer. Slight differences in topographical distribution of PrP^d ^accumulation were observed between the BSE affected deer: while five of the i.c. inoculated animals showed abundant PrP^d ^deposits in forebrain areas, the single orally-challenged and one of the i.c. inoculated animals accumulated PrP^d ^predominantly in the brainstem and cerebellum, and very little elsewhere. This last animal (deer 1 in table 1) was killed early in the clinical phase of disease for welfare reasons, and this may account for the lower overall magnitude of PrP^d ^and for the little involvement of forebrain areas. In contrast to the BSE infected deer, the brains of CWD-affected animals showed weak and inconsistent, fine punctate intra-neuronal labelling mainly in neurons of the brainstem and of the deep nuclei of the cerebellum. Like BSE cases, peri-neuronal labelling was conspicuous in the DMNV; unlike BSE cases, particulate and coalescing PrP^d ^labelling in the neuropil was prominent, with plaque like accumulations seen throughout most brain areas, often associated with white matter bundles and areas of conspicuous vacuolation (Fig. 3).

**Figure 4:**
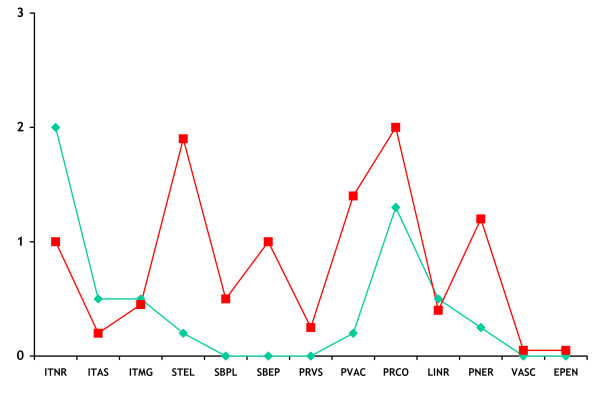
**IHC profile of PrP^d ^types present in BSE infected deer (green) and CWD infected deer (red)**. Profiles represent a single deer from each of the BSE and CWD infected groups. All six of the deer challenged i.c. with BSE and the single positive orally dosed deer presented consistent profiles on subjective examination that were unlike those of the CWD infected deer. (ITNR, intraneuronal; ITAS, intra-astrocytic; ITMG, intra-microglial; STEL, stellate; SBPL, sub-pial; SBEP, sub-ependymal; PVRS, peri-vascular; PVAC, peri-vacuolar; PRCO, particulate coalescing; LINR, linear; PNER, perineuronal; VASC, vascular; EPEN, Ependymal).

**Figure 5:**
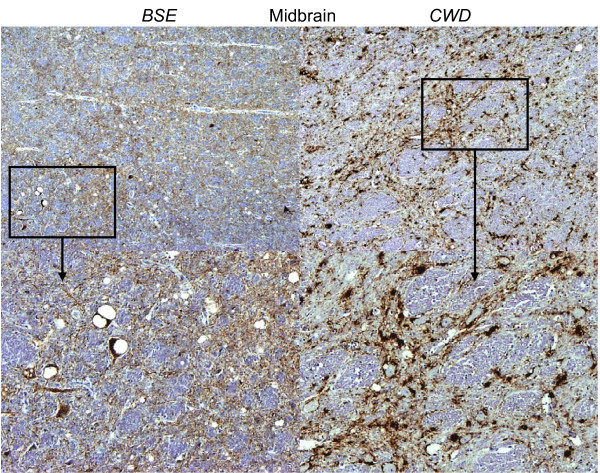
**BSE and CWD deer**. Low magnification images (top) of midbrain highlight the marked differences seen between the predominantly intra-neronal and difuse particulate labelling of the neuropil that is seen in the BSE infected deer compared to the distinct coalescing plaque-like labelling in the CWD infected deer. The boxes outline the area shown at higher magnification in the lower two images.

**Figure 6:**
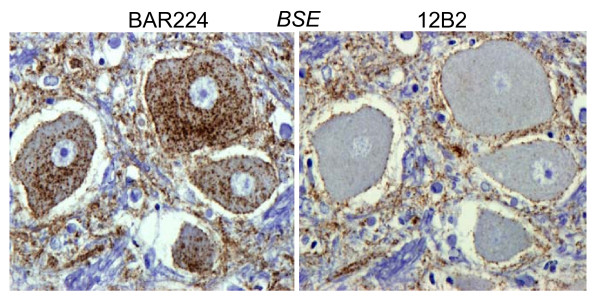
**BSE deer**. Comparison of intraneuronal labelling with BAR224 (C-terminal) and 12B2 (n-terminal) mAbs showing the significant loss of intraneuronal labelling with 12B2 in BSE infected deer in contrast to CWD infected deer. The extracellular signal was maintained with 12B2 in both the CWD and BSE infected deer.

**Figure 7:**
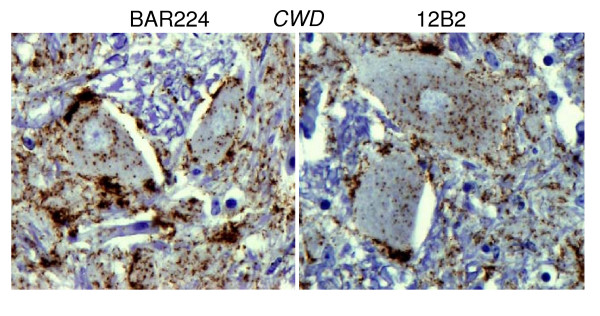
**CWD deer**. Comparison of intraneuronal labelling with BAR224 (C-terminal) and 12B2 (n-terminal) mAbs showing no reduction of the intracellular signal with the n-terminal targeting antibody.

These differences in the patterns of PrP^d ^accumulation between BSE and CWD resulted in distinct PrP^d ^profiles that made subjective discrimination relatively straightforward (Figs. 4, 5). Further differences were evident when evaluating the epitope mapping features of the PrP^d ^molecules of both infections. Samples of obex and midbrain were immunolabelled with N-terminal 12B2 antibody to assess the magnitude of intra-neuronal PrP^d ^signal in comparison with that obtained with BAR224. In BSE infected deer, the intra-neuronal PrP^d ^observed in almost all neuronal populations with BAR224 was significantly reduced or lost with 12B2 (Fig. 6), while in CWD affected animals, the lower level of intra-neuronal PrP^d ^labelled with BAR224 was nevertheless maintained with 12B2 (Fig. 7). No significant difference in extra-cellular signal recovery was seen between CWD and BSE when comparing results with these N-terminal and globular domain antibodies.

#### Accumulation of PrPd outside the brain

**Figure 8:**
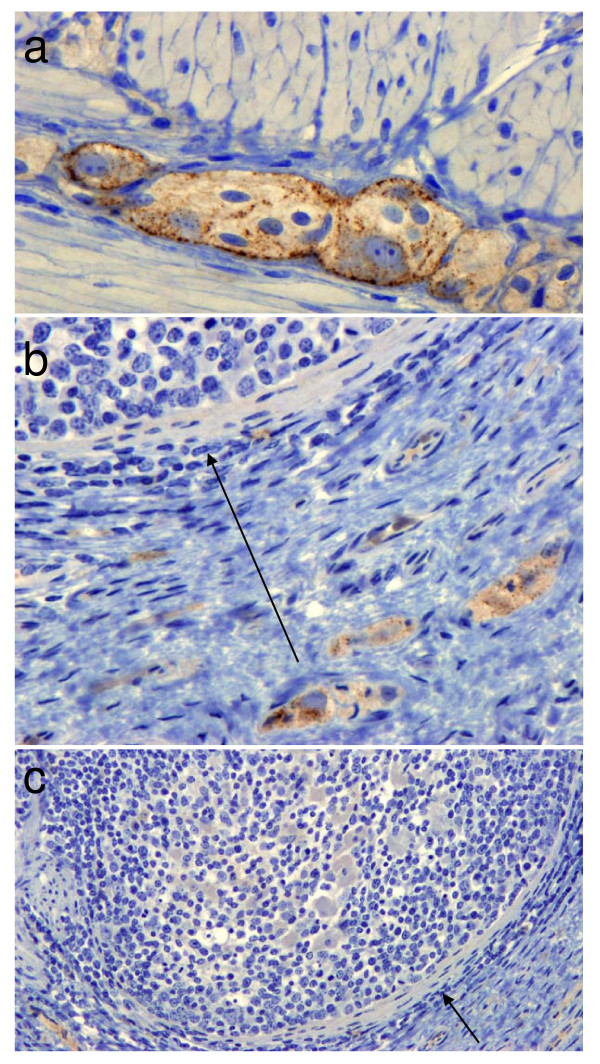
**BSE deer**. Labelling of PrP^d ^in cells of the myenteric (a) and sub-mucosal plexi (b) occurred in close proximity to negative Peyer's patches shown at high (b) and low magnification (c). Arrows show the capsule surrounding the same follicle.

**Figure 9:**
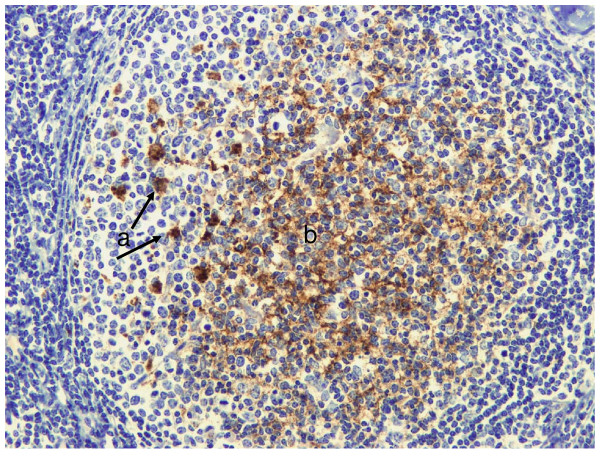
**CWD Deer**. Labelling of PrP^d ^in tingible body macrophages (a) and follicular dendritic cells (b) within the secondary follicle of a retropharyngeal lymph node.

In BSE inoculated deer, PrP^d ^was detected in all segments of the spinal cord, in autonomic ganglia, cranial and peripheral nerves and in the sensory retina. Strong immunolabelling was found in neurones throughout the enteric nervous system, sometimes in close proximity to nearby lymphoid follicles (Fig 8). However, the Peyer's patches, and all other lymphoid tissues, were negative, as were all other organs examined (for details see [8]). In contrast, CWD infected deer were consistently PrP^d ^positive in rectal biopsies and in the retropharyngeal lymph node (Fig 9).

### Western blotting

**Figure 10:**
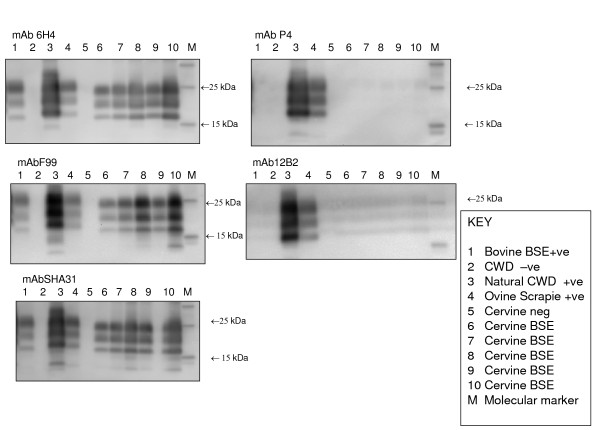
**BSE deer**. Analysis of brain homogenates by Biorad TeSeE Western Blotting using F99, 6H4, SHA31, P4 and 12B2. Data is not shown for the last of the intracerebrally challenged group or for the single orally challenged BSE positive deer however these presented migration patterns and antibody affinities that were not significantly different from those seen above.

**Figure 11:**
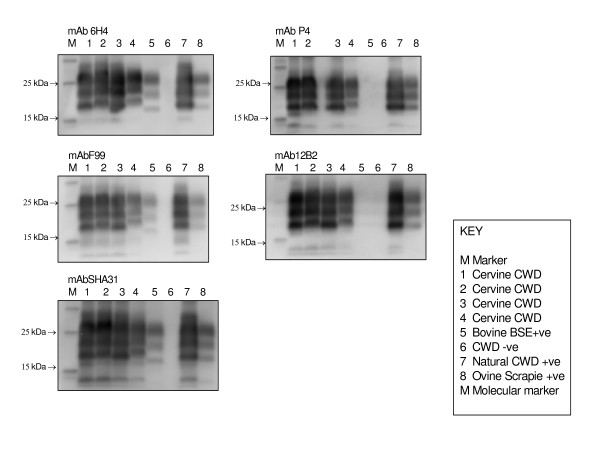
**CWD deer**. Analysis of brain homogenates by Biorad TeSeE Western Blotting using F99, 6H4, SHA31, P4 and 12B2. The molecular weights of samples from the four red deer experimentally infected with CWD were variable although all of them appeared higher than the cattle BSE control and are more consistent with the naturally infected CWD and scrapie controls.

The samples from red deer experimentally infected with BSE, either orally or i.c., all gave molecular weight profiles similar to those of cattle BSE, with a lower molecular weight for the unglycosylated protein band compared to that obtained for ovine scrapie, elk CWD or red deer CWD samples; the latter showed some degree of variability. The antibody affinity was also different between BSE and CWD infected deer. The former showed strong signal with antibodies raised to the C-terminal region of the prion protein (F99) and to globular domain (6H4 and Sha31) and no signal with the antibodies raised to the N-terminus of PrP (P4 and 12B2; Fig. 10), while this reactivity was present in CWD infected animals (Fig. 11).

## Discussion

Differences in attack rates and incubation periods observed between oral cattle BSE (16%), intracerebral cattle BSE (100%) and oral deer CWD (100%) included in this study suggest that there is a strong species barrier between cattle and deer in the case of BSE. Although it needs to be pointed out that the remaining five deer orally dosed are still alive and that infection cannot be ruled out, this species barrier would agree with the low attack rate found in intracerebral infections of cattle with the CWD agent [24].

The examinations conducted on this experimental material show that infections of red deer with BSE and at least one source of CWD can be clearly differentiated by the same laboratory methods previously used to discriminate between BSE and scrapie in sheep [19,25,26] and goats. Thus, the IHC epitope mapping approach shows that intra-neuronal PrP^d ^in BSE infected deer is truncated at an epitope further towards the C terminus of the PrP molecule than it does in CWD. This differential truncation also takes place when exogenous enzymes are applied to brain samples as indicated by the discriminatory WB results.

As for the subjectively assessed patterns of IHC PrP^d ^accumulation -the PrP^d ^profile-, while these were highly consistent amongst BSE infected deer, some variability was observed in the CWD cases, with one deer showing a slightly divergent profile to the other three and also a considerably higher magnitude PrP^d ^signal in all brain areas. This variability was also observed in the electrophoretic migration speed of PrP^res ^in the red deer CWD samples, as previously described for elk, mule deer and white tail deer [27], raising the possibility that more than one phenotype of CWD exists even amongst a limited number of animals. Further work is required to investigate whether the IHC profile and molecular weight variation seen in the red deer CWD samples has any significance regarding strain diversity, or is related to other as yet unidentified influences. We were able to examine the obex but not other brain areas of each of the four elk that provided the pooled inoculum for the CWD infected red deer, and the distribution and patterns of PrP^d ^observed in those samples were indistinguishable from those in the obex of the CWD red deer recipients. Similarly, all four elk used in the inoculum demonstrated almost identical molecular profiles, in contrast with the CWD recipient red deer where some variability was observed. It is thus possible that the host species may have an effect on molecular and pathological phenotypes.

The minor differences in the topographical distribution of PrP^d ^found between the BSE orally and i.c. challenged deer may be due to differences in the route of access of infectivity to the brain, as proposed for sheep infected with BSE [12]. In this model, it has been shown that PrP^d ^accumulates in the porencephalic lesion resulting from the repair process at the site of inoculation, and appears to spread to other neighbouring areas of the cerebral cortex [28]. This pathway would be absent in orally dosed animals, explaining the lesser involvement of rostral areas of the brain.

The fact that the circumventricular organs, which have leaky capillaries and appear to be involved in the neuroinvasion process associated with infectivity present in blood [29], were affected in these BSE infected deer adds support to this notion.

PrP^d ^was detected in the enteric nervous system and in structures of the peripheral nervous system of the BSE infected deer, regardless of the route of challenge. It is difficult to tell whether that peripheral PrP^d ^accumulation preceded neuroinvasion or resulted from centrifugal spread, but the lack of LRS involvement in those same deer rather supports the later possibility. This is unlikely to be due to the i.c. route of challenge, as the only orally-dosed deer succumbing to BSE was also negative for PrP^d ^in lymphoid tissues, and as lymphoid involvement has been shown in BSE i.c. challenged sheep of several *PRNP *genotypes [30]. The absence of LRS involvement seen in BSE-affected red deer is more likely to respond to host genetic factors, as it is reminiscent of cattle BSE, in which the only documented evidence of LRS accumulation of PrPd refers to Peyer's patches after experimental oral challenge [31], and of experimental BSE in sheep bearing the ARR *PRNP *allele [29,30].

The absence of PrP^d ^in LRS tissues of BSE affected deer, as in natural cattle BSE, might be indicative of a low level of circulating infectivity and suggests that BSE in deer may not be contagious under conditions of natural exposure. Conversely, red deer infected with CWD showed widespread lymphoid involvement, which would be in agreement with the highly contagious nature of this infection in native North American cervids.

## Conclusion

European red deer are susceptible to infection with the cattle BSE agent, not only by the intra-cerebral but also by the oral route, and although the clinical signs and spongiform change are similar to those of CWD in the same species, these two infections can be easily differentiated. The lack of lymphoid involvement, the PrP^d ^truncation pattern both "*in vivo*" and "*in vitro*", and the predominantly intracellular accumulation of PrP^d ^are features of deer BSE that are in contrast with those of deer CWD. However, only one of six deer developed disease after alimentary exposure to 25 g of a BSE brain pool homogenate after an incubation period of nearly 5 years; this suggests a strong species barrier but if a TSE in European red deer should ever be identified then BSE/CWD discrimination would be an urgent priority. To determine whether there are potential naturally occurring BSE-like strains and to determine the degree to which there is strain variation, it would be necessary to examine many more naturally occurring CWD cases. These results will support the ongoing European surveillance for natural TSEs in red deer and the further assessment of potential risk to human health.

## Authors' contributions

SM collated the experimental data and wrote the paper. MJ, LG and AB designed the study. SM, SS, LG, MJ and AB examined the tissues. MS, MC and PS performed the biochemical analyses. MD and HR challenged, managed and monitored the deer. All authors have read and approved the final manuscript.
